# Assessing the impact of short-term Lugol’s solution on toxic nodular thyroid disease: a pre-post-intervention study

**DOI:** 10.3389/fendo.2024.1420154

**Published:** 2024-07-25

**Authors:** Fredric Hedberg, Per Karkov Cramon, Robert Bränström, Henrik Falhammar, Jan Calissendorff

**Affiliations:** ^1^ Department of Endocrinology, Karolinska University Hospital, Stockholm, Sweden; ^2^ Department of Molecular Medicine and Surgery, Karolinska Institutet, Stockholm, Sweden; ^3^ Department of Clinical Physiology and Nuclear Medicine, Copenhagen University Hospital – Rigshospitalet, Copenhagen, Denmark; ^4^ Department of Endocrinology, Copenhagen University Hospital – Rigshospitalet, Copenhagen, Denmark; ^5^ Department of Breast, Endocrine Tumors and Sarcoma, Karolinska University Hospital, Stockholm, Sweden

**Keywords:** Lugol, iodine, toxic adenoma, toxic nodular goiter, thyrotoxicosis, hyperthyroidism

## Abstract

**Purpose:**

Preoperative iodine therapy in toxic nodular goiter (TNG) is discouraged as iodine may cause aggravation of hyperthyroidism. We aimed to examine if a short course of iodine treatment is safe to administer in TNG.

**Methods:**

Patients with TNG (n=20) and subclinical to mild hyperthyroidism (free (f)T4 <30 pmol/L) without complicating illnesses were included in this pre-post-intervention study at Karolinska University Hospital. All participants received Lugol’s solution 5%, three oral drops thrice daily for 10 days. Heart rate, TSH, fT4, fT3 concentrations were collected before (day 0) and after treatment (day 10). Thyroid hormone concentrations were also measured at two time points during treatment to discover aggravations of hyperthyroidism. ThyPRO39se, a quality-of-life questionnaire, was filled out day 0 and day 10. Differences in heart rate, thyroid hormone concentrations, and quality-of-life before and after treatment were compared. Adverse reactions were reported.

**Results:**

The median age was 63.5 years. Female to male ratio 19:1. FT4 and fT3 concentrations decreased (both *p*<0.001), and TSH concentration increased (*p*<0.001) after 10 days of treatment. There was no difference in heart rate. No aggravations of thyrotoxicosis were noticed in any of the participants. ThyPRO39se scores improved on three scales, including hyperthyroid symptoms, while the remaining scale scores were unchanged. Mild and transient symptoms related to or possibly related to treatment were observed in six participants.

**Conclusion:**

A short course of Lugol’s solution improved thyroid hormone concentrations, reduced patient-reported hyperthyroid symptoms and was safe in TNG. Lugol’s solution might be an option for preoperative treatment in TNG.

**Clinical trial registration:**

https://www.clinicaltrials.gov, identifier NCT04856488.

## 1 Introduction

Iodine has been used as preoperative treatment of hyperthyroidism in Graves’ disease for more than a century (1). At that time, the American endocrinologist Dr Henry Plummer found that the surgical outcome after thyroidectomy due to Graves’ disease was significantly improved, and mortality decreased after preoperative treatment with Lugol’s solution (1). A hundred years ago, clinical assessment was used to evaluate hyperthyroidism (2). Today, we also have reliable laboratory tests (3). Since the introduction of antithyroid drugs and radioiodine treatment, iodine preparations before surgery have been used more sparsely (4).

However, American and European guidelines for hyperthyroidism still recommend iodine as an add-on treatment to antithyroid drugs before surgery due to Graves’ disease (5, 6). Iodine has been shown to reduce thyroid perfusion (7) and have positive effects on surgical complications such as transient hypoparathyroidism (8). When surgery due to hyperthyroidism is planned, the recommendation is to render the patient euthyroid before surgery (9). This is believed to reduce the risks of aggravated thyrotoxicosis in uncontrolled hyperthyroidism associated with anesthesia and in the postoperative period (9).

On the other hand, administration of iodine preparations before surgery due to toxic nodular goiter (TNG) is explicitly not recommended (5). The evidence for this recommendation is based on epidemiological studies on iodine fortification programs, and the risk of hyperthyroidism after administration of iodine-containing radiology contrast media (10). It is unclear whether these findings support the recommendation against a short course of Lugol’s solution in preparation for thyroidectomy due to TNG.

Based on this lack of evidence, we aimed to perform an explorative intervention study on the short-term effects of 10 days of Lugol’s solution on patients with TNG. This study challenges the current perception that iodine should not be used in preparation for surgery for toxic nodular thyroid disease.

## 2 Materials and methods

### 2.1 Ethics

This study was performed in line with the principles of the Declaration of Helsinki and conducted following ICH-GCP guidelines, including external monitoring. The Swedish Ethical Review Authority approved the study. Written informed consent was obtained from all subjects. The study intervention and protocol were approved by The Swedish Medical Products Agency (EudraCT 2019 – 002242 – 21) and registered at ClinicalTrials.gov (ID: NCT04856488).

### 2.2 Setting and study design

An experimental pre-post-intervention study was conducted between November 2021 and February 2023 at the Department of Endocrinology at Karolinska University Hospital in Stockholm, Sweden. The Department of Endocrinology at Karolinska University Hospital is a tertiary referral center for endocrinology and a quaternary care center for radioiodine treatment of benign thyroid disease in the Stockholm metropolitan area. Sweden is considered iodine-sufficient (11).

### 2.3 Participants and procedures

Patients with toxic nodular thyroid disease, referred from primary and secondary care centers in Stockholm, were asked to participate in this study. Inclusion criteria were age 18 to 75 years, clinical diagnosis of TNG with persistent subclinical to mild hyperthyroidism (suppressed thyroid-stimulating hormone (TSH) in combination with free T4 (fT4) and free T3 (fT3) within reference ranges, or elevated fT4 <30 pmol/L), and negative TSH receptor antibodies. Exclusion criteria were previous thyroid surgery, unstable coronary heart disease, congestive heart failure NYHA III-IV, chronic kidney disease stage 3-5, chronic liver disease Child-Pugh Score A-C, current infection, ongoing glucocorticoid, amiodarone, or anticoagulant treatment, thyroid eye disease CAS >2p, diabetes mellitus type 1, active cancer, severe psychiatric illness, pregnancy, breastfeeding, fertile woman without contraceptives, and iodine hypersensitivity. Mental incapacity, unwillingness, or language difficulties which lead to difficulties understanding the meaning of participation were also exclusion criteria.

All patients were treated with oral drops of Lugol’s solution 5%, consisting of iodine 5 g, potassium iodide 10 g and 85 g water. The dose was three drops three times a day for 10 days, summing up to 603 mg of iodine. Ingestion of ≥80% of the prescribed doses was considered compliant.

Blood samples for TSH, fT4, and fT3 concentrations were measured before and after treatment (day 0 and day 10). These tests were also collected on day 3 or 4, and day 6 or 7 to identify exacerbations of hyperthyroidism during treatment. The quality of life (QoL) questionnaire ThyPRO39se (12, 13) was completed in paper form at the Department of Endocrinology before and after treatment (day 0 and day 10). The investigators subsequently entered the QoL data into a REDCap database. Blood samples were analyzed using electrochemiluminescence, Roche Cobas, at the Karolinska University Hospital laboratory. Reference intervals were: TSH 0.3 - 4.2 mIU/L; fT4 12 - 22 pmol/L; and fT3 3.1 - 6.8 pmol/L.

Ingested doses of Lugol’s solution and adverse reactions were recorded in a patient diary. Adverse reactions were assessed for severity and whether they were related to the intervention.

Subsequent clinical follow-ups and patient management were performed at the Department of Endocrinology at Karolinska University Hospital in Stockholm, Sweden.

### 2.4 ThyPRO39, patient-reported quality-of-life questionnaire

ThyPRO is a disease-specific patient-reported outcome questionnaire developed for benign thyroid diseases (14). ThyPRO39 is the short form (12) comprising 39 items on physical, mental, and social domains of functioning and well-being. ThyPRO39 is available in several linguistically and qualitatively validated translations, including the Swedish ThyPRO39se (13). The items are summarized in 13 scales (goiter, hyperthyroid, hypothyroid, eye, and tiredness symptoms, cognitive complaints, anxiety, depressivity, emotional susceptibility, impaired social life, impaired daily life, cosmetic complaints, and overall quality of life impact) as well as a composite score from the seven well-being and functioning scales. Each item employs a recall period of four weeks and is rated on a 0 to 4 Likert scale from 0 = “no symptoms/problems” to 4 = “severe symptoms/problems”. The item scores are summarized in scale scores as described elsewhere (12). Higher scores indicate worse health status.

### 2.5 Outcome measures

The primary outcome was changes from baseline in thyroid hormone concentrations and heart rate after 10 days of treatment with Lugol’s solution. Secondary outcomes were changes in patient-reported QoL scale scores of ThyPRO39se. Adverse reactions were assessed if associated with the intervention and by severity and duration.

### 2.6 Statistical analysis

All statistical analyses were performed using Stata/BE version 17.0. Descriptive data were summarized as medians and interquartile ranges (IQR). Categorical variables were reported as frequencies (n) and percentages (%). Due to the small sample size, the assumption of normal distributions was not met.

Differences between pre- and post-tests for thyroid hormone concentrations, heart rate and ThyPRO39se scales were analyzed using the non-parametric Wilcoxon signed-rank test for paired data. *P*-values less than 0.05 were considered significant. The study was an exploratory pilot study, and no à priori power calculation was made.

## 3 Results

### 3.1 Patient characteristics

**Figure 1:**
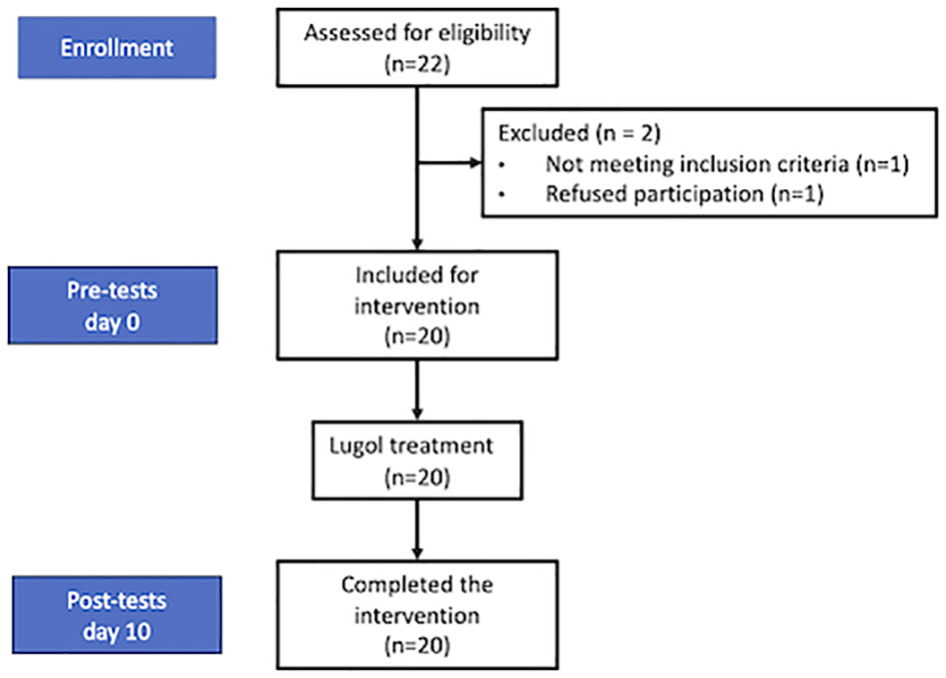
Flow-chart of the participants in the pre-post-intervention study.

A total of 22 patients were interested and screened for eligibility. Twenty patients were included and informed consents were obtained. One patient did not meet the eligibility criteria and another refused participation after screening (**Figure 1**).

The median age was 63.5 years (IQR 56-68), and 19 (95%) were female. One patient (5%) was treated with an antithyroid drug (Thiamazole), and 8 (40%) were on beta-blocker treatment. These medications remained unchanged during the study period.

Baseline medians (IQR) of thyroid hormone concentrations were TSH 0.07 (0.02-0.2) mIU/L, fT4 17.0 (15.5-19.0) pmol/L, and fT3 5.4 (5.1-5.9) pmol/L. The median (IQR) heart rate was 72 beats per minute (56–68) (**Table 1**).

**Table 1:** Clinical and laboratory baseline data of patients with toxic nodular goiter.

Variable	Lugol day 0 n = 20	
Female, n (%)	19	(95%)
Age in years, median (IQR)	63.5	(56–68)
Heart rate (BPM), median (IQR)	72	(56–68)
TSH (mIU/L), median (IQR)	0.07	(0.02-0.2)
Free T4 (pmol/L), median (IQR)	17.0	(15.5-19.0)
Free T3 (pmol/L), median (IQR)	5.4	(5.1-5.9)
β-blocker treatment, n (%)	8	(40%)
Antithyroid drug treatment, n (%)	1	(5%)

Reference ranges: TSH 0.3 - 4.2 mIU/L, free T4 12 - 22 pmol/L, free T3 3.1 - 6.8 pmol/L.

IQR, interquartile range; BPM, beats per minute.

All patients completed the study, although one patient ingested only 77% of the prescribed doses of Lugol’s solution.

### 3.2 Thyroid hormones and heart rate

**Figure 2:**
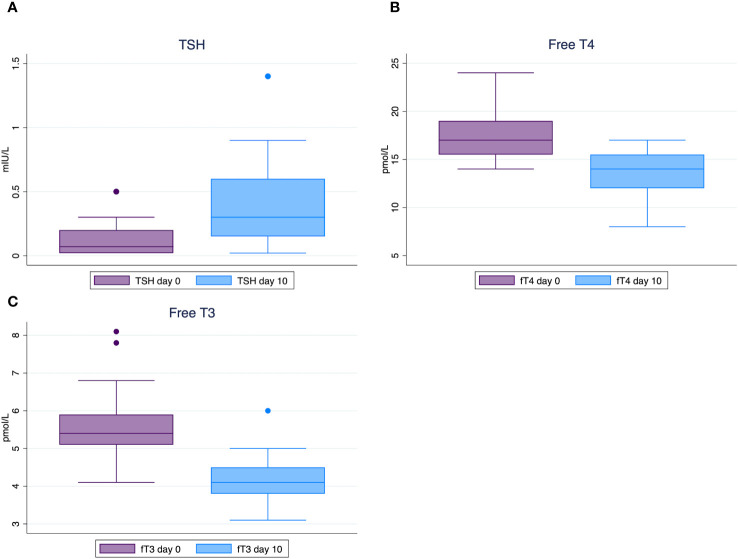
Box plots of thyroid hormone concentrations showing medians and interquartile ranges before and after 10 days of Lugol’s solution in toxic nodular goiter. TSH concentrations **(A)**; Free T4 concentrations **(B)**; Free T3 concentrations **(C)**. Reference ranges: TSH 0.3 - 4.2 mIU/L, free T4 12 - 22 pmol/L, free T3 3.1 - 6.8 pmol/L.

Significant differences in median changes pre- vs post-treatment (Lugol day 0 vs Lugol day 10) were found for TSH (0.07 vs 0.3 mIU/L, *p <*0.001), fT4 (17 vs 14 pmol/L, *p <*0.001) and fT3 (5.4 vs 4.1 pmol/L, *p <*0.001). All participants showed a milder biochemical state of hyperthyroidism with increased TSH, and lower fT4 and fT3 compared to baseline. The clinical status measured as heart rate remained unchanged before vs after treatment with Lugol’s solution (**Table 2**, **Figure 2**).

**Table 2:** Thyroid hormone concentrations and heart rate before and after treatment for patients with toxic nodular goiter.

Variable	Lugol day 0		Lugol day 10		*P*-value	Reference
	n = 20		n = 20			range
	Median	(IQR)	Median	(IQR)		
Heart rate (BPM)	72	(56–68)	73	(68–79)	0.11	
TSH (mIU/L)	0.07	(0.02-0.2)	0.3	(0.15-0.6)	<0.001	0.3-4.2 mIU/L
Free T4 (pmol/L)	17	(15.5-19)	14	(12-15.5)	<0.001	12-22 pmol/L
Free T3 (pmol/L)	5.4	(5.1-5.9)	4.1	(3.8-4.5)	<0.001	3.1-6.8 pmol/L

BPM, beats per minute; TSH, thyroid-stimulating hormone.

**Figure 3:**
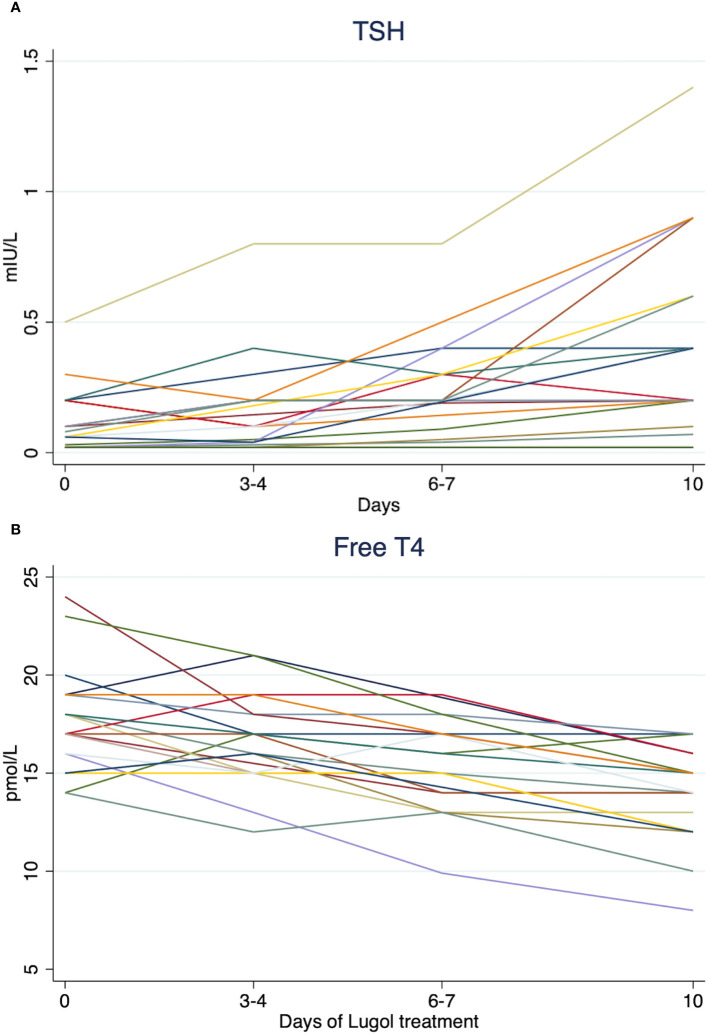
Spaghetti plots of TSH **(A)** and free T4 **(B)** concentrations for all patients with toxic nodular goiter during treatment with Lugol’s solution at day 0, day 3-4, day 6-7, and 10. Reference ranges: TSH 0.3 - 4.2 mIU/L, free T4 12 - 22 pmol/L, free T3 3.1 - 6.8 pmol/L.

None of the patients had aggravation of thyrotoxicosis during treatment (**Figure 3**).

### 3.3 ThyPRO39se

After treatment, median scale scores had improved (decreased) significantly on two of the 13 individual scales and the composite scale, compared to baseline measurements. The patients reported an improvement in median (IQR) scores before vs after treatment in hyperthyroid symptoms (25.5 (18-30.5) vs 18 (8–23), *p* 0.03) and less depressive symptoms (29 (14–41) vs 18 (14–33), *p* 0.008) as well as a reduced median (IQR) composite score (22.5 (13-39.5) vs 18.5 (9-26.5), *p* 0.007), indicating better well-being and functioning. The remaining scale scores were unchanged (**Table 3**).

**Table 3:** ThyPRO39se scale scores for patients with toxic nodular goiter (n=20) included in the study on Lugol’s solution.

Scale	ThyPRO39se					
	Scale range	Baseline		Follow-up		*P*-value
	Min-max	Median	(IQR)	Median	(IQR)	
Goiter symptoms	2–84	2	(2–17.5)	2	(2–15)	0.76
Hyperthyroid symptoms	2–90	25.5	(18–30.5)	18	(8–23)	0.026
Hypothyroid symptoms	0-100	19	(6–31)	13	(6–22)	0.063
Eye symptoms	1–89	14	(1–29)	4,5	(1–25)	0.27
Tiredness symptoms	0–100	42	(29–75)	42	(25–62.5)	0.18
Cognitive complaints	1–95	14	(1–33)	14	(4–21)	0.39
Anxiety	1–96	18	(5.5–37.5)	18	(1–26)	0.12
Depressive symptoms	0–97	29	(14–41)	18	(14–33)	0.0078
Emotional susceptibility	1–95	24.5	(10–44)	13	(10–40)	0.29
Impaired social life	0–100	0	(0–17)	0	(0–4)	0.41
Impaired daily life	0–98	3.5	(0–26)	0	(0–15)	0.17
Cosmetic complaints	1–96	1	(1–12)	1	(1–12)	0.81
Overall quality of life impact	0-100	25	(0–37.5)	25	(0–37.5)	1.00
Composite	0-100	22.5	(13–39.5)	18.5	(9–26.5)	0.0067

IQR, interquartile range; Min-max, minimum to maximum.

### 3.4 Adverse reactions

Out of six participants, representing 30% of the total, adverse reactions possibly linked to the treatment, such as gastrointestinal symptoms and headache, were reported. All symptoms were classified as mild and transient.

Following the study intervention, participants were enrolled in the routine clinical care for patients with TNG. Thyroid scintigraphies were conducted, including evaluations for possible radioiodine treatment. Multinodular disease was observed in 18 cases (90%), while solitary adenomas were detected in two cases (10%).

## 4 Discussion

This study investigated the short-term effects of 10 days of Lugol’s solution in 20 patients with TNG with subclinical to mild thyrotoxicosis, and it is to our knowledge the first that prospectively studies iodine treatment solely in TNG. Treatment with Lugol’s solution resulted in an increase in TSH and a decrease in fT4 and fT3 concentrations. None of the patients suffered any exacerbations of thyrotoxicosis during the intervention. Along with reducing biochemical thyrotoxicosis, improved ThyPRO39se scale scores for hyperthyroid symptoms reinforced the results. No deteriorations in QoL scale scores were observed.

Nearly half of the patients (40%) were on treatment with a stable dose of beta-blockers, which effectively reduce heart rate. However, the heart rate remained unaltered, supporting the conclusion that no aggravation of thyrotoxicosis occurred during Lugol’s treatment.

Several case series on iodine treatment in hyperthyroidism published during the 1900s included patients with TNG. These studies point towards an immediate beneficial effect on basal metabolic rate and thyroid hormones in the first week of iodine treatment in thyrotoxic patients, regardless of the cause of hyperthyroidism (2, 15–21). The applied doses varied between 1 mg to 390 mg iodide daily and durations varied from one to 10 weeks. Different preparations of iodine and different methods of outcome measurement methods were used in these studies.

Over time, epidemiological studies on iodine fortification programs have revealed an increased incidence of hyperthyroidism in the years following the initiation of fortification (22). This, in addition to the Jod-Basedow effect, or aggravation of hyperthyroidism seen after administration of iodine contrast media has raised concerns about the use of iodine, since patients with TNG as well as patients with Graves’ disease seem particularly susceptible to this side effect (10, 23–25). The current recommendations that discourage using iodine as preoperative treatment in TNG are based on the fear of iodine-induced hyperthyroidism (5, 10). The results from our study contradict this perception.

It should be noted that the long-term daily dosage in μg of iodine in fortification programs (26), and exposure of between 15-37 g iodine from a single dose radiology contrast (25) differ substantially from the clinical use of iodine preparations in Graves’ disease (4) and the doses used in our study.

The findings in this study indicate that Lugol’s solution might be considered a short-course treatment to improve thyroid hormone status before thyroidectomy in patients with TNG. Particularly, it may be a relevant alternative in cases when there are contraindications for antithyroid drugs.

From a surgical standpoint, Lugol’s solution has been a longstanding preoperative intervention in the management of Graves’ disease. While the exact mechanism remains elusive, its endorsement is echoed across European and North American guidelines. This study sheds further light on the efficacy of Lugol’s solution, thereby potentially elucidating its rationale for preoperative use. However, in Graves’ disease, the benefits extend beyond hormonal modulation. Emerging evidence suggests that preoperative Lugol administration not only normalizes hormonal concentrations, but also affects thyroid vascularity and blood perfusion, thereby reducing the complexity of the surgery and associated risks (7, 8, 27–30). Thus, while the hormonal equilibrium restoration remains a significant aspect, Lugol’s solution appears to offer multifaceted advantages in the preoperative treatment of Graves’ disease, although large prospective investigations are lacking.

The strengths of our study include the prospective pre-post-intervention design. The intervention drug has a fast onset of effect, and the duration of the intervention was short, which reduces the risks of confounding due to seasonal variations, other treatments, or other exogenous factors. The main limitations were a small sample size and no control group. Although the counterfactual scenario is assumed to be an unchanged thyrotoxic status, results from the ThyPRO39se should be interpreted with caution, as well as assessments of adverse reactions. Another limitation was the low external validity from the outcomes restricted to a single institution in an iodine-sufficient area, and a sample without concurrent complicating illnesses. Furthermore, the study did not report treatment response beyond the study intervention, such as effects on thyroid hormone concentrations past 10 days of treatment.

In conclusion, our prospective intervention study demonstrates the safety and efficacy of a short course of Lugol’s solution, evidenced by improvements in biochemical markers and QoL assessments. The observed adverse reactions were mild and transient, suggesting the tolerability of Lugol’s solution in this context. These findings indicate that Lugol’s solution may represent a viable option when a short course treatment is warranted immediately before thyroidectomy for patients with TNG.

Further studies, preferably randomized controlled clinical trials are warranted to validate these findings, particularly in cohorts with larger sample sizes, comprising detailed physical examinations, patients with comorbidities and more severe thyrotoxicosis. Additionally, the potential impact of preoperative Lugol’s solution on facilitating surgery and surgical outcomes in TNG remains an intriguing area for future investigation.

## Data availability statement

The raw data supporting the conclusions of this article will be made available by the authors, without undue reservation.

## Ethics statement

The studies involving humans were approved by The Swedish Ethical Review Authority. The studies were conducted in accordance with the local legislation and institutional requirements. The participants provided their written informed consent to participate in this study. Written informed consent was obtained from the individual(s) for the publication of any potentially identifiable images or data included in this article.

## Author contributions

FH: Conceptualization, Data curation, Formal analysis, Investigation, Visualization, Writing – original draft, Writing – review & editing. PC: Data curation, Resources, Writing – review & editing. RB: Supervision, Writing – review & editing, Conceptualization. HF: Funding acquisition, Resources, Supervision, Writing – review & editing, Project administration. JC: Conceptualization, Investigation, Project administration, Supervision, Writing – review & editing.
